# Dual-stage segmentation and classification framework for skin lesion analysis using deep neural network

**DOI:** 10.1177/20552076251351858

**Published:** 2025-07-13

**Authors:** Khadija Manzoor, Nauman U Gilal, Marco Agus, Jens Schneider

**Affiliations:** 1 College of Science and Engineering, 370593 Hamad Bin Khalifa University, Doha, Qatar; 2 Qatar Computing Research Institute, 370593 Hamad Bin Khalifa University, Doha, Qatar

**Keywords:** Skin lesion segmentation, deep learning, image augmentation, skin disease classification, skin cancer

## Abstract

**Objective:**

Skin diseases, caused by various pathogens including bacteria, viruses, and fungi, are prevalent globally and significantly affect patients’ physical, emotional, and social well-being. Early and accurate detection of such conditions is critical to prevent progression, especially in cases of malignant skin lesions. This study aims to develop a dual-stage deep learning framework for the segmentation and classification of skin lesions, addressing challenges such as imbalanced data, lesion variability, and low contrast.

**Methods:**

We propose a two-phase framework: (i) Precise instance segmentation using U-Net with a Visual Geometry Group (VGG16 encoder) to isolate skin lesions and (ii) classification using EfficientFormer and SwiftFormer networks to evaluate performance on both balanced and imbalanced datasets. Experiments were conducted on three benchmark datasets: Human against machine with 10,000 training images (HAM10000), International Skin Imaging Collaboration (ISIC) 2018, and the newly released ISIC 2024 SLICE-3D dataset. For SLICE-3D, we evaluated both tabular-only and image + metadata fusion approaches using XGBoost classifier and ResNet-based classifier, respectively.

**Results:**

On the balanced HAM10000 dataset, EfficientFormerV2 achieved 97.11% accuracy, a 97.14% *F*_1_-score, 96.85% sensitivity, and 96.70% specificity. On the ISIC 2018 dataset, the segmentation model achieved 97.59% accuracy, 89.12% Jaccard index, and 94.24% Dice similarity coefficient. For the ISIC 2024 SLICE-3D challenge, the tabular-only XGBoost classifier achieved a partial area under the receiver operating characteristic curve score of 0.16752, while the image + tabular fusion model achieved a score of 0.15792 using ResNet, demonstrating competitive performance in a highly imbalanced and clinically realistic setting.

**Conclusion:**

The proposed dual-stage deep learning framework demonstrates high accuracy and robustness across segmentation and classification tasks on diverse datasets. Its adaptability to large-scale, non-dermoscopic data such as SLICE-3D confirms its potential for deployment in real-world skin cancer triage and teledermatology applications.

## Introduction

The incidence of cancer is on the rise, driven by factors such as harmful behaviors like smoking and alcohol consumption. Other significant contributors include environmental changes, various types of radiation exposure, viral infections, poor dietary habits, and unhealthy lifestyle choices.^1^ Among the different types of cancer, skin cancer stands out as one of the most prevalent and dangerous. Skin cancer typically manifests as abnormal growths or swellings of skin cells. This disease is not only spreading rapidly across the globe but also poses a significant health threat.^2^ Each year, the United States reports approximately 5.4 million new cases of skin cancer, emphasizing the critical importance of developing effective prevention and treatment strategies.^3^ Therefore, skin diseases, including skin cancer, are a significant and growing global health concern. According to the International Agency for Research on Cancer World Health Organization (WHO), melanoma, a prevalent form of skin cancer, caused 324,635 new cases and 57,043 related deaths in 2020.^4^ These figures are projected to increase to 500,000 new cases and 96,000 deaths by 2040,^5^ placing melanoma 15th among the most prevalent cancer types. Early diagnosis is crucial to prevent the progression of melanoma to metastatic stages, affecting other organs and tissues. Recognizing and addressing skin lesions in a timely manner is vital for patient survival. Because of the resemblances among various types of skin cancer, even seasoned dermatologists might struggle to categorize them correctly. Nonetheless, research indicates that proficient dermatologists can visually identify skin cancer with an accuracy ranging from 75% to 80% by utilizing the asymmetry, border irregularity, color variation, diameter guideline, which evaluates asymmetry, border, color, and diameter.^6–8^ Diagnostic discrepancies can arise from factors such as low image resolution, varying expertise, and visual impairments. Computer-based diagnostic systems can mitigate these issues by aiding in the classification and diagnosis of skin lesions.^9–11^ The International Skin Imaging Collaboration (ISIC) datasets, spanning from 2016 to 2020,^12–17^ have played a pivotal role in revolutionizing skin cancer diagnosis by offering extensive and carefully annotated image collections. These comprehensive datasets have paved the way for groundbreaking applications of machine learning and deep learning technologies in dermatology,^9,18–22^ empowering the development of advanced computer-aided diagnostic systems that support dermatologists in detecting and categorizing skin lesions with enhanced accuracy and diagnostic consistency.^9,10,23^

**Figure 1.**
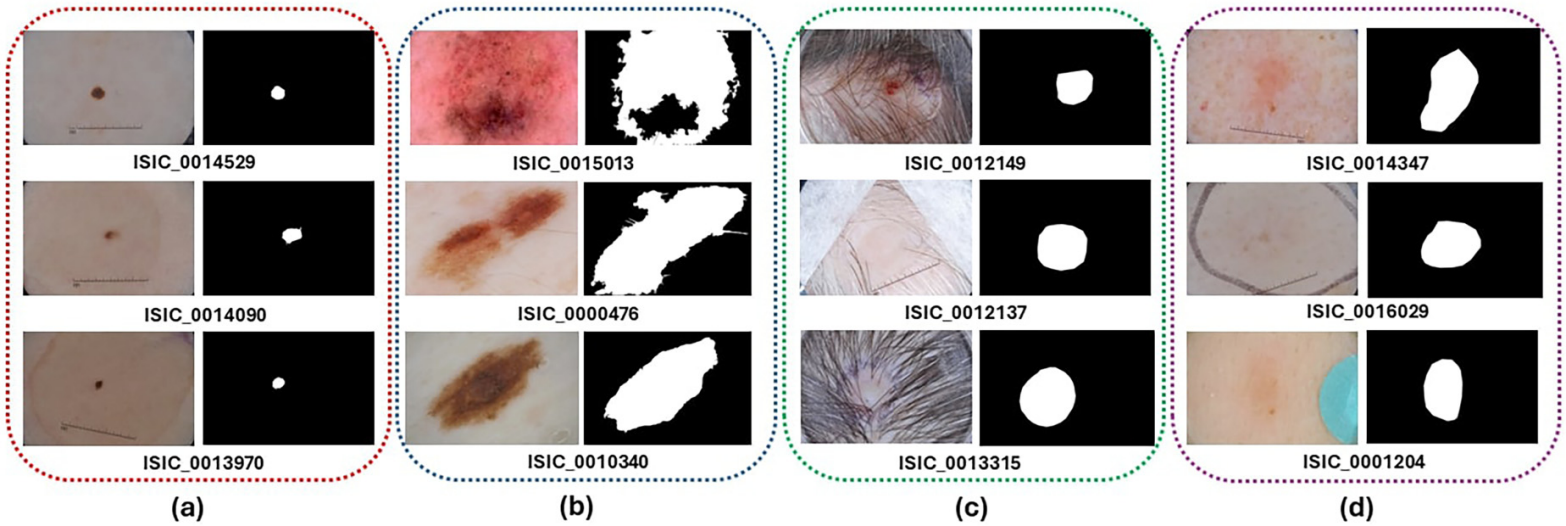
Challenges in skin lesion images from the International Skin Imaging Collaboration (ISIC)-2018 dataset: (a) Very small skin lesions; (b) lesions with unusual shapes; (c) images with defects or unclear parts; (d) lesions blending with background color.

Skin cancer diagnosis applications benefit from image preprocessing, feature extraction, and accurate segmentation of the diseased area to enhance segmentation and classification accuracy. In this research, we examine the ISIC-2018 skin cancer dataset^14^ to carry out segmentation and classification tasks. Figure 1 demonstrates the notable differences in the appearance and dimensions of skin lesions. The automatic segmentation and classification of skin lesions presents a difficult challenge due to various factors. Dermatologists take dermoscopic images that differ in size, shape, contour, and color distribution. Moreover, the presence of hair, blood vessels, and other artifacts further complicates the segmentation and classification of skin lesions for computational systems. In contrast, a significant challenge with the ISIC datasets lies in the data's inherent imbalance, where certain skin lesion categories are underrepresented compared to others. This disparity can potentially generate biased models that perform exceptionally well in majority classes while demonstrating limited effectiveness for minority classes. Addressing this complexity demands the strategic implementation of advanced data augmentation techniques, sophisticated balanced sampling approaches, and robust machine learning algorithms to ensure comprehensive and accurate classification across diverse skin lesion^9,21^ types. In the categorization of skin disorders, medical experts recognize two main types. Melanotic lesions develop from melanocytes, the cells that generate skin pigment, resulting in abnormal pigmentation patterns.

**Figure 2.**
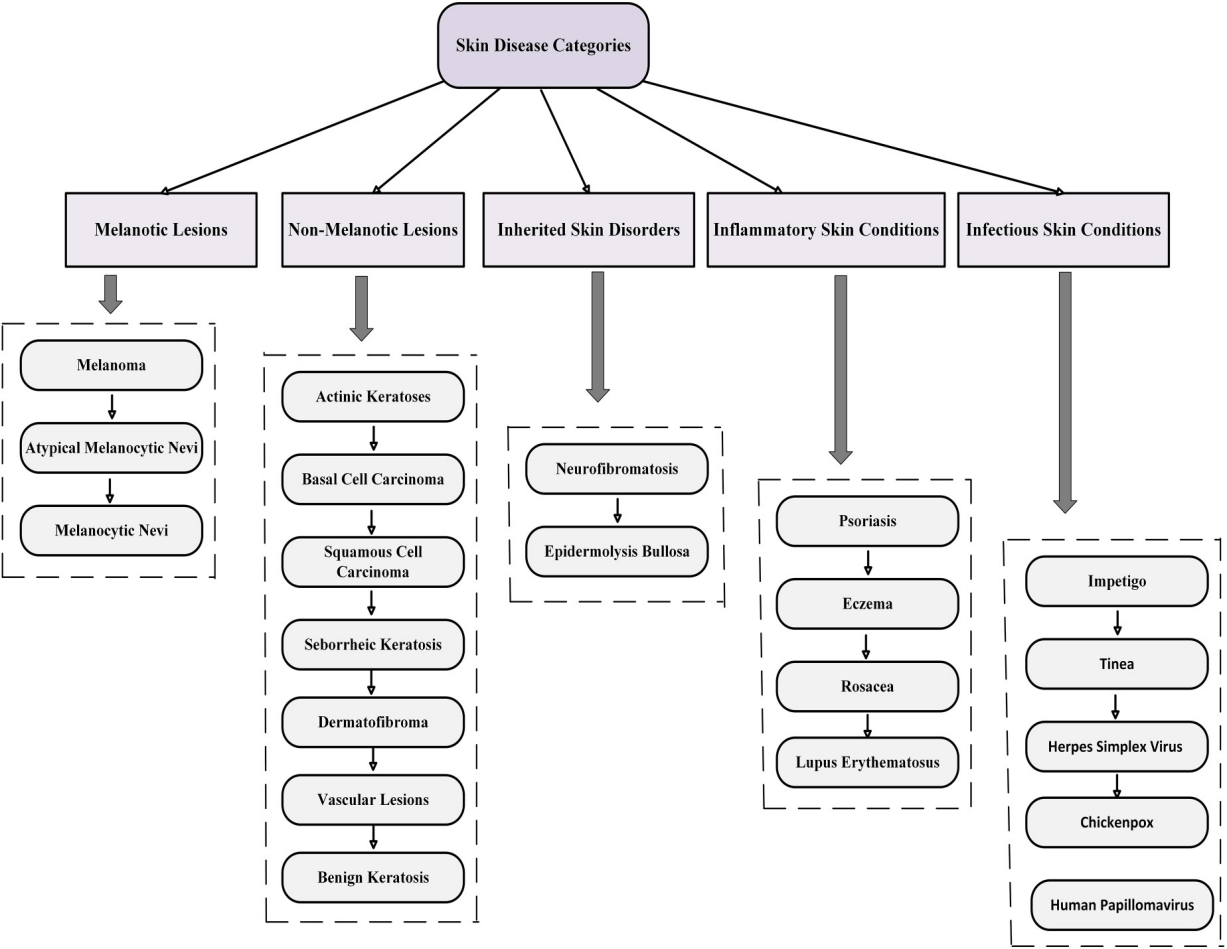
A classification chart of skin disorders, categorized into melanotic and non-melanotic lesions, as well as other key groups.

Conversely, non-melanotic lesions originate from different skin cells or structures, not involving melanocytes. Medical practitioners rely on this essential distinction to establish suitable diagnoses and treatment approaches. Figure 2 illustrates a detailed classification of skin diseases that emerge from various other skin components. The diagram organizes skin diseases into five primary categories: Melanotic lesions, non-melanotic lesions, inherited skin disorders, inflammatory skin conditions, and infectious skin conditions. Each category is further divided into specific types, including melanoma under melanotic lesions and psoriasis under inflammatory skin conditions. This systematization aids in comprehending the variety and intricacy of skin-related disorders, providing a framework for diagnosis and treatment.

Despite the numerous segmentation and classification methods proposed to address these challenges,^4,21,23–28^ significant gaps remain in achieving consistent skin lesion segmentation. To bridge these gaps, we introduce a comprehensive framework that starts with an instance segmentation component, which is crucial for accurately extracting skin lesions from images and isolating them from the background. This step is pivotal for effectively analyzing the specific characteristics of each lesion. We also incorporate a deep-learning-based classifier, which significantly enhances lesion classification from dermoscopic images.

### Motivation

Skin cancer poses a significant public health challenge, and timely detection is essential for reducing mortality rates. Even with the growing application of dermoscopy, manual assessment of lesions often leads to variable and subjective outcomes. Although deep learning approaches have demonstrated potential for automating the classification of lesions, datasets such as human against machine with 10,000 training images (HAM10000) are missing segmentation masks, which diminishes the accuracy of lesion isolation. This research addresses this issue by utilizing U-Net alongside a Visual Geometry Group (VGG16 encoder) (trained on ISIC 2018) to extract lesion patches from the images in HAM10000. EfficientFormerV2 and SwiftFormer are then employed to classify the segmented lesions, thereby enhancing diagnostic precision and providing a scalable solution for real-time clinical implementation. The key contributions of our framework are:

- Dual-stage segmentation and classification framework: This article introduces a deep learning framework consisting of two phases for the automated evaluation of skin lesions, which combines segmentation and classification to enhance diagnostic precision. A U-Net model utilizing a VGG16 encoder was developed to isolate lesion areas in dermoscopic images, trained on the ISIC 2018 dataset. The identified lesions are then classified using EfficientFormerV2 and SwiftFormer, two advanced lightweight transformer models that were trained on the HAM10000 dataset. By employing segmentation prior to classification, lesions are distinguished from the background, thereby reducing interference and improving the accuracy of classification.
- Balanced and imbalanced classification: The classification models, EfficientFormerV2 and SwiftFormer, are trained using both balanced and imbalanced datasets to ensure stable performance across lesion classes with varying frequencies. Balanced datasets are generated through augmentation to address class imbalance, while imbalanced datasets reflect actual clinical scenarios. This combined training approach enhances the model's capability to accurately identify both rare and common lesion types, facilitating broader use in clinical environments.
- Dual-dataset integration for real-world simulation: The proposed pipeline employs two separate datasets, ISIC 2018 for segmentation and HAM10000 for classification, to mimic actual dermatological workflows. Segmentation enhances the borders of lesions before classification, ensuring that EfficientFormerV2 and SwiftFormer concentrate solely on the areas of the lesions. This integration enhances diagnostic accuracy by bridging the gap between lesion discovery and classification in automated systems.
- Innovative use of EfficientFormerV2 and SwiftFormer in dermatological practices: This study presents the application of EfficientFormerV2 and SwiftFormer for the classification of skin lesions, showcasing their effectiveness in medical image analysis. By integrating lightweight architectures with the efficiency of transformers, the models provide scalable, high-performance options suitable for real-time healthcare and telemedicine environments. This research highlights the effectiveness of transformer-based models in dermatological diagnosis, which deliver improved accuracy and computational efficiency compared to standard convolutional neural network (CNN)-based methods.

## Related work

This section critically reviews recent studies pertinent to our research, systematically organized around key thematic domains: The U-Net architectural framework and its variant approaches, innovative strategies for data balancing, sophisticated classification methodologies, and the strategic implementation of mobile inference in skin lesion diagnostic analysis.

### U-Net architecture and variants

The U-Net architecture has become a cornerstone for medical image segmentation due to its precision in capturing fine details. U-Net^29^ is particularly renowned in image semantic segmentation, notably within medical imaging. It utilizes a conventional encoder-decoder framework in which the encoder incorporates pooling layers for downsampling, while the decoder employs deconvolution or bilinear interpolation for upsampling. During this procedure, the spatial and edge details from the original images are progressively reconstructed, transforming low-resolution feature maps into segmentation masks at the pixel level. To mitigate spatial information loss during downsampling, U-Net concatenates features from corresponding encoder and decoder positions, thereby enhancing the resolution and detail restoration during upsampling.

Subsequent models have built upon the U-Net framework to further enhance biomedical image segmentation. M-Net^30^ incorporates a multi-scale U-shaped convolutional network featuring a side output layer designed for multi-label segmentation. U-Net++^31^ introduces nested, dense skip pathways for improved feature fusion. AttU-Net^32^ incorporates attention gates to highlight salient features. Additionally, MultiResUNet^25^ offers several enhancements to the original U-Net, while MFSNet^21^ applies boundary and reverse attention modules for refined segmentation outputs. Despite these advances, many redesigned skip connections still do not fully leverage multi-scale feature interactions. To address this, the MSB module is designed to enhance multi-scale feature utilization through improved interaction.

MSCA-Net^23^ have showcased enhanced diagnostic precision by leveraging multi-scale contextual data in medical imaging. Assessments on datasets such as ISIC 2017, ISIC 2018, and PH2 indicate that MSCA-Net outperforms other leading methods in the segmentation of skin lesions. Recent developments in medical image segmentation, such as DLGRAFE-Net^33^ and LightCF-Net^34^ have demonstrated advanced segmentation capabilities in gastrointestinal polyp detection. While these approaches focus on polyp segmentation, their architectural innovations can inspire improved lesion analysis in dermatology. In this study, we employed the U-Net architecture^29^ with a VGG16 encoder to carry out skin lesion segmentation on the ISIC 2018 dataset.

### Deep and machine learning techniques

Early melanoma detection using smartphones faces constraints such as memory and battery limitations. This study develops a system that analyzes images in seconds and diagnoses melanoma stages using different features. The dataset contains 184 images, including 67 malignant melanoma and 117 benign nevi, segmented using Otsu and minimum spanning tree methods. Features like border, color, texture, and asymmetry are extracted with gray-level co-occurrence matrix and local binary patterns (LBPs), and normalized mutual information feature selection selects relevant features for classification.^35^ The researchers presented an advanced adaptive fine-tuned CNN that is intended to automatically distinguish between skin tumors that are melanoma and those that are not. By replacing traditional fully connected layers with a two-phase transfer learning strategy that incorporates lesion-specific regions of interest (ROIs) and principal component analysis (PCA), their method successfully reduces model complexity and reduces the chance of overfitting. Through the use of adaptive max pooling and PCA-processed ROIs to reconfigure the initial convolutional layer, this technique enhances model generalization for dermoscopic images of different sizes. Results from experiments on benchmark datasets, such as HAM10000, showed a high classification accuracy, under-scoring the method's substantial potential for application in clinical settings for early skin cancer screening and diagnosis.^36^ A new hybrid model has been introduced that integrates VGG19 with the network-in-network structure to address the limitations of conventional CNN architectures in skin cancer detection. This design significantly reduces the number of trainable parameters while enhancing feature extraction through micro-networks and nonlinear transformations. By employing global average pooling and transfer learning, the model demonstrates improved convergence and generalization across various lesion sizes. It achieved an accuracy rate of 90% for both benign and malignant classifications when evaluated on the HAM10000 dataset. Due to its strong performance and computational efficiency, this model presents a practical option for use in clinical settings.^37^ The multi-lesion classification is a challenging task in skin cancer detection. The authors compared five deep learning methods to classify six main skin diseases: Actinic keratosis (AK), Rosacea, basal cell carcinoma (BCC), squamous cell carcinoma (SCC), Lupus Erythematosus, and seborrheic keratosis (SK). The methods compared include ResNet50, Inception V3, DenseNet-121, Xception, and Inception-ResNet V2. The Inception-ResNet V2 model achieved the best results. A dataset containing 2656 facial images was established for these six diseases. The authors also performed studies using an independent dataset from other body parts and conducted transfer learning on their models. The dataset used for multiclass classification problems included 4006 training images and 388 test images.^38^ Moreover, another study on skin cancer, which remains a challenging task in the fields of deep learning and machine learning.^39^ The authors used deep learning and machine learning algorithms to classify skin cancer. The proposed method discriminates between malignant and benign skin lesions to ensure appropriate treatment for the patient. Deep learning algorithms (VGG16, AlexNet, and ResNet-18) as well as machine learning algorithms (support vector machine (SVM)) were used. Two disease classes were considered: melanoma and SK. The dataset was gathered from the ISIC, containing 2037 images of the aforementioned diseases. The proposed fully automatic method achieved an accuracy of approximately 90.69% for skin lesion classification. Dermoscopic skin tumor analysis is one of the most difficult tasks in skin cancer detection, primarily due to the challenge of differentiating cancerous lesions, which is particularly difficult for even experienced dermatologists. There are various types of skin cancers that are harmful to people, and detecting these cancers manually is not feasible. However, melanoma is considered the deadliest and most aggressive form of skin cancer.^40^ An effective approach to diagnose melanoma using machine learning techniques has been proposed. The dataset was collected from the PH2 database, which contains a total of 200 images: 40 melanomas, 80 atypical nevi, and 80 common nevi. The aim of this research is to detect both non-melanoma and melanoma skin lesions from dermoscopic images.

### Classification methods

Various classification methods have been proposed to enhance skin lesion diagnosis. Brinker et al.^41^ compared the performance of a CNN trained to detect melanoma in dermoscopic images to the diagnoses provided by dermatologists. Using 12,378 dermoscopic images, they highlighted the potential of CNNs to match expert performance. Putri et al.^42^ utilized LBP features and Näıve Bayes classifiers for classifying skin diseases, finding good accuracy on small datasets. Their work addressed the classification of nine classes, covered by the ISIC and HAM10000 datasets. Harangi^43^ developed an ensemble model to classify melanoma, nevus, and SK. By merging the outputs of five independent deep NN architectures (GoogleNet, AlexNet, ResNet, VGGNet) using various ensemble techniques, they achieved high classification accuracy. Kassem et al.^44^ developed a computerized system that employs CNNs and transfer learning methods to classify eight categories of skin conditions, such as benign keratosis, SCC, vascular lesion, dermatofibroma, AK, melanocytic nevus, melanoma, and BCC. Methods in computer vision have been utilized to improve the effectiveness of analyzing skin lesions by classifying dermoscopic images. Employing the HAM10000 dataset, which contains 10,015 dermatoscopic images, the research assessed a variety of advanced deep-learning models. The research implemented four advanced CNNs: InceptionV4, SENet154, InceptionResNetV2, and PNASNet-5-Large. Among these architectures, PNASNet-5-Large demonstrated superior performance in discriminating between different types of skin lesions.^45^ This research addresses the challenges related to the automatic classification of skin lesions, focusing particularly on the issue of substantial visual resemblance between different types of lesions. The study utilizes CNNs and transfer learning methods, especially incorporating the GoogleNet architecture. The evaluation includes three extensive datasets: ISIC 2019, HAM10000, and BCN 20000. While GoogleNet yielded encouraging outcomes, the research pointed out critical limitations related to computational demands, especially when attempting to utilize more intricate architectures like VGG19,^44^ which created hardware accessibility issues for researchers across different regions According to Table 1, recent research on the diagnosis of skin cancer through deep learning presents diverse methodologies, datasets, and results over multiple years.

**Table 1.** Summary of most recent literature on skin cancer detection using deep learning.

Ref	Problem	Dataset	Techniques	Pros	Cons	Results
2024^46^	Skin lesion	HAM10000	CNN with NLM denoising and sparse dictionary learning	Improved accuracy, Noise reduction	Computationally heavy, no lesion segmentation	HAM10000: 85.61%
						ISIC-2019: 81.23% AUC: 0.977
2024^47^	Skin cancer	MNIST-HAM10000	Stacked CNN (with data augmentation)	Outperforms standard models.	High computational cost, Sensitive to noise	HAM10000: 96%
		ISIC-2020 (33,126 images)				ISIC-2020: 73%
2024^48^	Multi-class skin lesion classification	HAM10000 (10,015 images)	ALBEF, DenseNet121, InceptionV3, ResNet50	Reduce overfitting, Multimodal learning	Limited metadata used, complex model architecture, imbalanced dataset	ALBEF: 94.11%
						AUROC: 0.9426
2022^49^	Multi-class skin cancer classification	HAM10000 (10,015 images) clinical dataset (1016 images)	ViT, Multi-scale patch, contrastive learning	Multi-scale embedding, robust feature extraction	High memory use, limited clinical validation	HAM10000: 94.3%
						Clinical: 94.1%
2022^50^	Skin lesion classification	ISIC 2018	InceptionV3, ResNet50, Custom CNN	Effective preprocessing (ESRGAN), Transfer learning, Data augmentation	High computational cost, risk of overfitting, limited dataset diversity, class imbalance	Accuracy: 85.7% (InceptionV3), AUC: 0.92%
2020^51^	Skin lesion classification	HAM10000 (10,015 images)	ResNet50, InceptionV3 MobileNet,	Low parameter count, lightweight model (MobileNet), fast inference time	Imbalanced dataset, Requires large computational power (ResNet50, InceptionV3)	ResNet50: 90%
						MobileNet: 87%
2022^52^	Multi-class lesion classification	PH2, DermIS, MEDNODE, ISIC2016, ISIC2017, ISIC2018	Refined RDCNN	Robust across datasets, overcomes class imbalance	Computationally intensive, High memory consumption	PH2: 94.97%
						ISIC2017: 96.29% 95.05% (ISIC2018)
2024^53^	Skin cancer classification (B/M)	ISIC2020 (33,126 images)	E-VGG19 + SVM, KNN, DT	Outperforms ResNet	Requires highquality images	Accuracy: 91%
2024^54^	Multiclass skin lesion classification	HAM10000, BCN-20000	ST, SwinV2-Large	High performance, captures spatial features	Complex modeling and computational cost	HAM10000: 94.3%,
						BCN: 85.7%
2024^55^	Multi-class skin cancer classification	Skin cancer: Malignant vs benign HAM10000	Skin-CAD (XAI + CNN)	Dual-layer feature extraction enhances performance	Complexity due to ensemble CNNs	HAM10000: 96.5%

ISIC: International Skin Imaging Collaboration; AUC: area under the receiver operating characteristic curve; ROC: receiver operating characteristic curve; RDCNN: residual deep convolutional neural network; SVM: support vector machine; KNN: K-nearest neighbor; XAI: explainable artificial intelligence; HAM10000: Human against machine with 10,000 training images; NLM: non-local means; ST: swin transformer.

Even with advancements in automated skin lesion detection, there are still difficulties in successfully combining segmentation and classification to improve diagnosis accuracy. The inability of several existing methods to seamlessly combine these two phases limits their usefulness in practical settings. Additionally, not enough balanced and imbalanced datasets have been explored, which is crucial for obtaining reliable results in clinical settings. Furthermore, even though dual-dataset integration replicates real dermatological operations, a major challenge still lies in effectively scaling these techniques to account for different skin types and lesion characteristics. Last but not least, transformer-based models have demonstrated promise in the interpretation of medical pictures; nevertheless, they still need to be improved in real-time, especially when telemedicine is involved. These gaps offer chances to enhance approaches and their application in real-world situations.

### Mobile inference for skin lesion analysis

The recent advent of mobile inference accelerators, paired with “Lite” versions of common deep learning APIs, has resulted in a surge in the computational power available for inference tasks on mobile devices such as smartphones. As a consequence, several authors have sought to exploit this new opportunity by proposing self-assessment or home pre-screenings of moles, opening a potential pathway to overcome the problem of performing public skin cancer screening at scale.^9,56^

## Methodology

**Figure 3.**
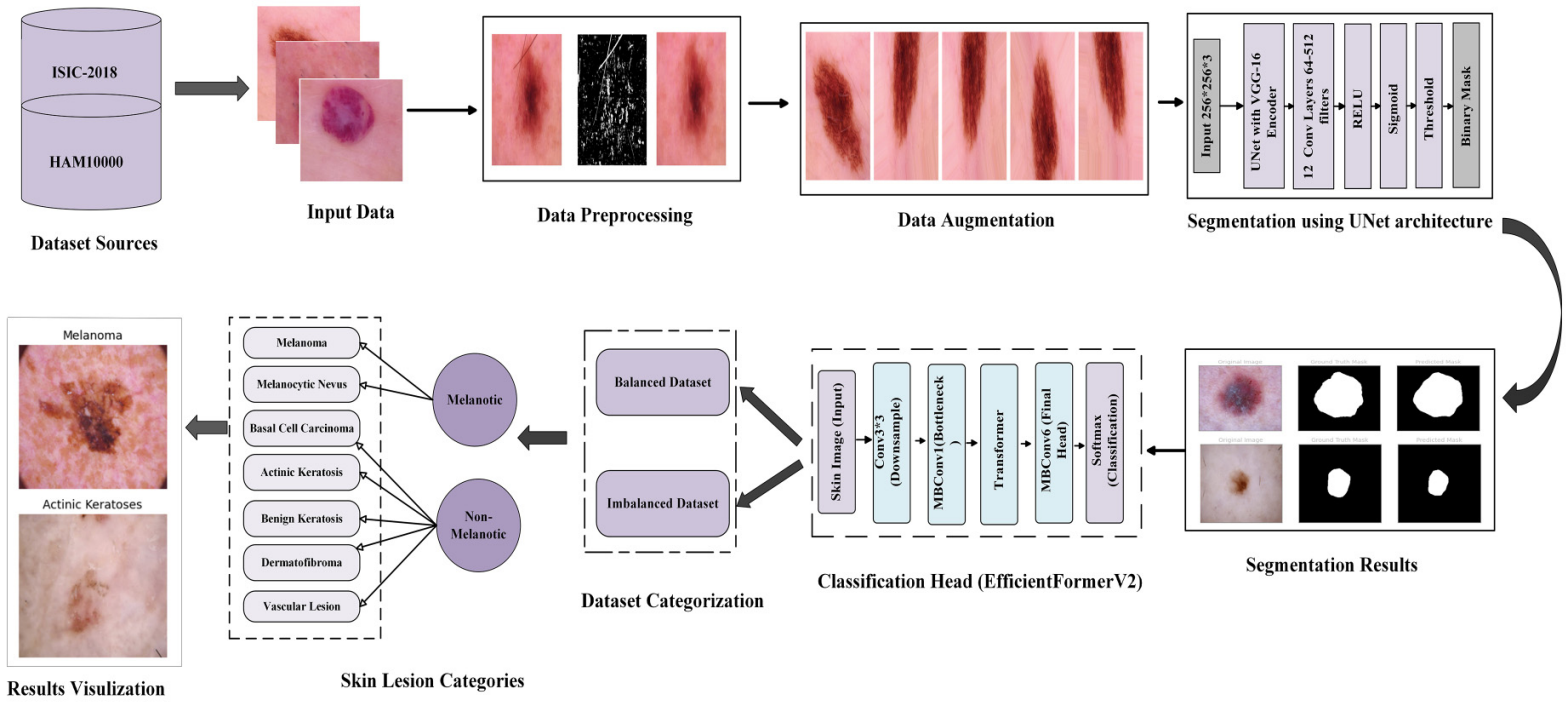
Overview of the proposed framework: The approach consists of three primary stages: (1) preprocessing of data, which includes resizing and removing hair, (2) augmenting the data through rotations and flips, and (3) analyzing lesions via U-shaped convolutional network (UNet) segmentation, subsequently classifying them into seven types of skin lesions using EfficientFormerV2.

This is a retrospective, experimental study conducted between **January 2023 and January 2025** at **Hamad Bin Khalifa University (HBKU), Qatar**. The objective was to develop and evaluate a dual-stage deep learning framework for skin lesion segmentation and classification using publicly available datasets. The study design involved the use of established benchmark datasets for segmentation (ISIC 2018^14^), classification (HAM10000^15^), and real-world binary malignancy prediction (SLICE-3D^57^). Figure 3 provides an overview of our framework's phases, which include classification, segmentation, image processing, and image augmentation. The process is shown in the diagram as it progresses from input data and preprocessing to augmentation techniques to address data imbalance. In order to achieve accurate skin lesion segmentation and classification, the last steps involve using U-Net for segmentation and EfficientFormerV2 for classification. In the following, we elaborate on each of these modules.

### Skin lesion datasets

In this work, we utilized three publicly available datasets: HAM10000,^15^ ISIC 2018,^13^ and the recently introduced SLICE-3D.^57^

#### HAM10000 dataset

The HAM10000 dataset consists of dermatoscopic images showcasing typical pigmented skin lesions collected from different populations. It includes seven diagnostic categories of skin lesions such as melanoma, melanocytic nevi, and BCC, among others. This dataset is widely used for multi-class classification tasks. In our study, we used HAM10000 to train a vision transformer (ViT) model for lesion classification. A summary of the class distribution is provided in Table 2. U-shaped convolutional network (UNet)^58^ The ISIC 2018 dataset^14^ is part of the ISIC Challenge series and includes dermoscopic images curated for three tasks: Lesion segmentation, lesion attribute detection, and disease classification. In this work, we focused on the segmentation task subset, which provides expert-annotated masks for binary segmentation of skin lesions that contains 2594 images and masks. A U-Net^58^ model with a VGG16 backbone was trained on this subset to delineate lesion boundaries. We summarized difference between both datasets explained in detail Table 3.

**Table 2.** Summary of Human Against Machine with 10,000 training images (HAM10000) dataset.

Category	Abbreviation	Number of images
Actinic keratoses	AKIEC	327
Basal cell carcinoma	BCC	514
Benign keratosis-like lesions	BKL	1099
Dermatofibroma	DF	115
Melanoma	MEL	1113
Melanocytic nevi	NV	6705
Vascular lesions	VASC	142
**Total**		**10015**

**Table 3.** Key differences between ISIC 2018 and HAM10000 datasets.

Feature	ISIC 2018	HAM10000
Primary task	Binary segmentation	Multi-class classification
No. of classes	1 (lesion vs. non-lesion)	7 lesion types
Objective	Delineate lesion boundaries	Identify lesion categories
Output format	Binary mask	Class label (e.g., melanoma)
Dataset size	2,594 images	10,015 images
Image type	Dermoscopic images	Dermoscopic and clinical images
Image resolution	Varies (e.g., 4288 × 2848 px)	Typically 600 × 450 px
Diversity	Multiple anatomical sites	Multiple lesion types
Annotation type	Expert segmentation masks	Expert class labels
Typical use case	Lesion segmentation research	Skin lesion classification
Preprocessing	Resizing, normalization	Resizing, normalization
Image sources	Clinical dermatology centers	Public dermatology repositories
Class balance	Relatively balanced	Imbalanced (dominated by nevi)

HAM10000: human against machine with 10,000 training images; ISIC: ISIC: International Skin Imaging Collaboration.

SLICE-3D dataset: The ISIC 2024 SLICE-3D dataset is a large-scale skin lesion dataset that contains 400,000 standardized lesion crops extracted from 3D total body photography (3D-TBP). The dataset was curated from seven dermatology centers across three continents and represents a significant advancement beyond traditional dermoscopic datasets. Each lesion image is paired with rich metadata describing patient demographics, anatomical site, lesion characteristics (e.g., size, asymmetry, hue, and border irregularity), and lighting conditions.

Images are categorized into:

- Strong-labels: Histopathology-confirmed malignant or benign lesions (biopsied within 3 months).
- Weak-labels: Clinically benign lesions assessed during full-body skin exams but not biopsied.

All images are standardized to a 15 × 15 mm field-of-view, with varying pixel resolutions. The classification task is binary: predicting the likelihood of malignancy for each lesion, making the dataset well-suited for real-world triage and early detection scenarios, especially in non-specialist or teledermatology settings. Compared to earlier ISIC datasets, SLICE-3D is uniquely:

- Non-dermoscopic, unlike prior dermoscopy-focused datasets.
- Massive in scale, including over 400k lesions from 1,000 + patients.
- Clinically representative, reducing selection bias by including all visible lesions per patient.
- Imbalanced, with only *≈* 0*.*1% malignant lesions, reflecting real-world clinical distributions.

### Image processing

**Figure 4.**
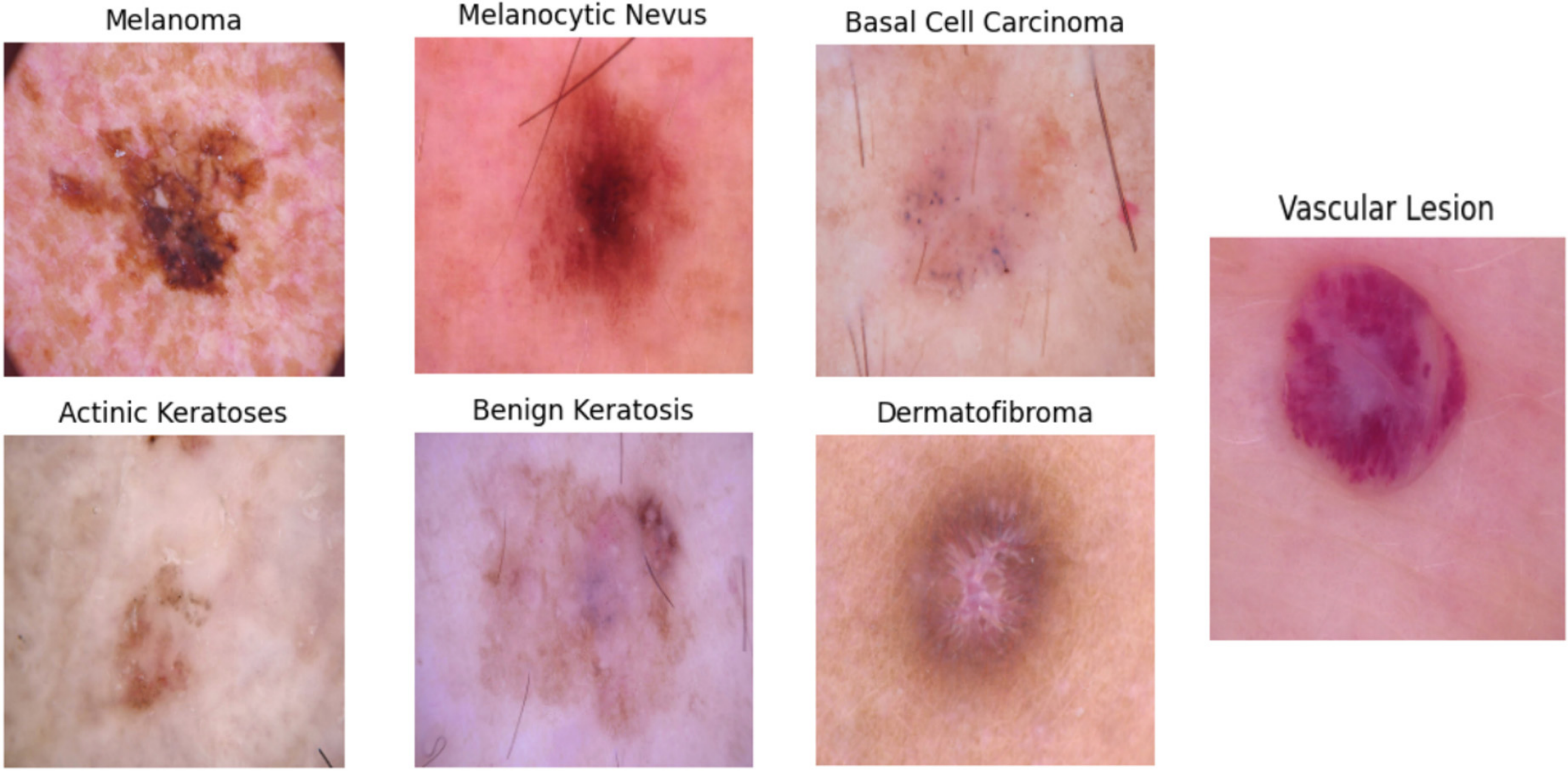
Examples for the lesion types discussed in this study. Notice the different color spaces across tissues and lesions.

**Figure 5.**
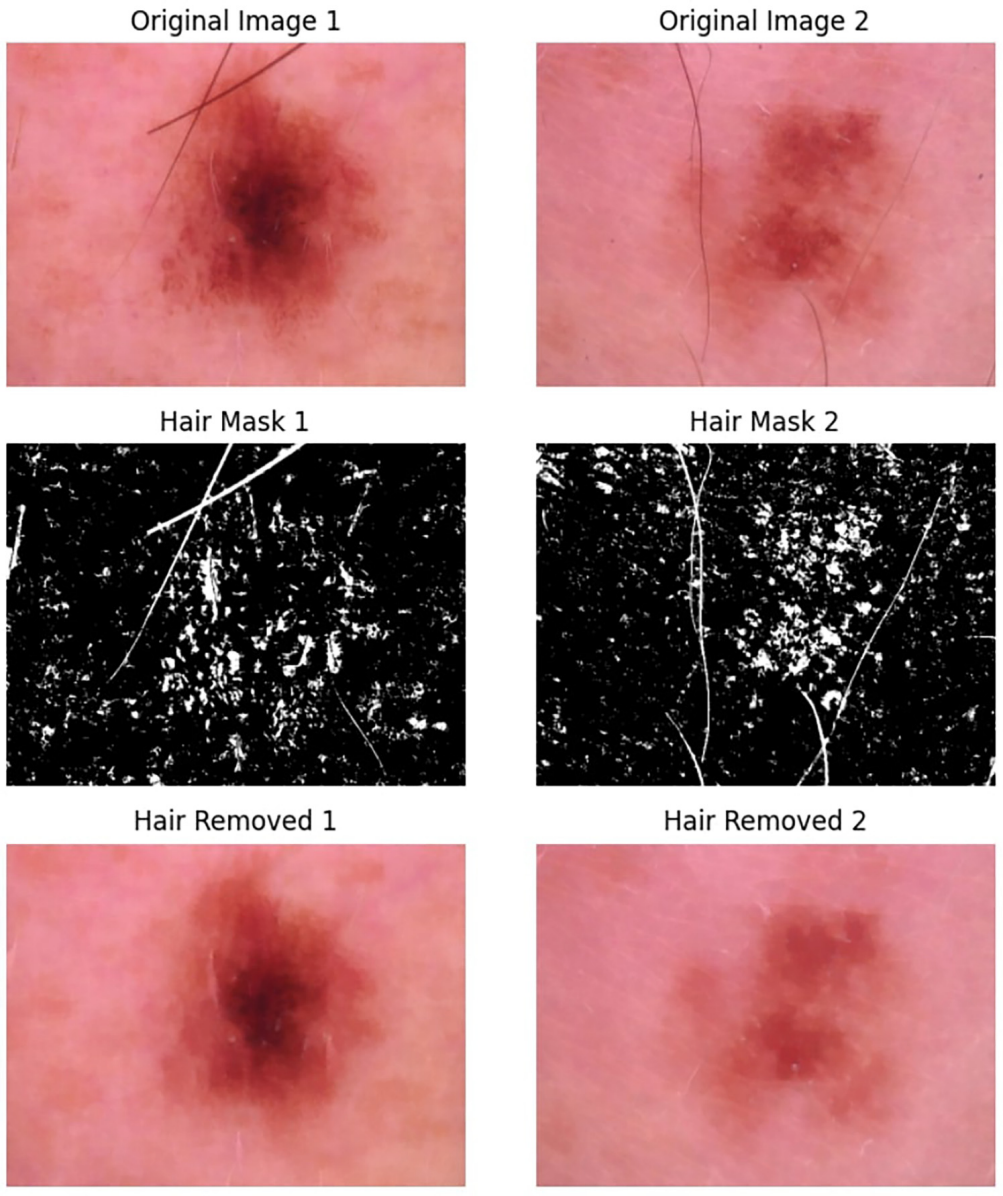
Three-stage hair removal process in dermoscopic images: original lesions with hair (top), detected hair masks using Blackhat filter (middle), and final hair-removed images via inpainting (bottom).

Our framework receives dermoscopic images as input. A common challenge with these images is that they can come in a wide range of color gamuts, hindering performance due to cross-domain generalization problems. While color equalization^59,60^ can alleviate this problem substantially, we found that converting images to a consistent color space followed by range normalization (in our case, mean-removal) is a computationally cheap and effective alternative(also see Figure 4 for colorspace variations ranging from reddish to light pink). Since image resolutions do not necessarily match model input resolutions, we rescale the images to 224 *×* 224 *×* 3. Again, to keep the framework as lightweight as possible. The hair removal algorithm utilizes a multi-step methodology that integrates morphological operations with inpainting techniques. A Blackhat morphological filter using a rectangular structuring element (17 × 17 kernel) is employed to differentiate dark hair structures from the background. This technique is particularly effective as it isolates dark features that are smaller than the defined kernel size. The resulting image, filtered by Blackhat, is then subjected to binary thresholding at a value of 10 to produce an accurate hair mask, successfully segmenting the hair structures from the lesion. Lastly, the algorithm applies the Telea inpainting method with a radius of 3 pixels to reconstruct areas obscured by hair. This method evaluates the surrounding pixel values to intelligently fill in the masked regions, preserving the natural texture and coherence of the skin lesion's features.In this research, we present the results of the hair removal procedure illustrated in Figure 5.

### Image augmentation to address imbalance

**Figure 6.**
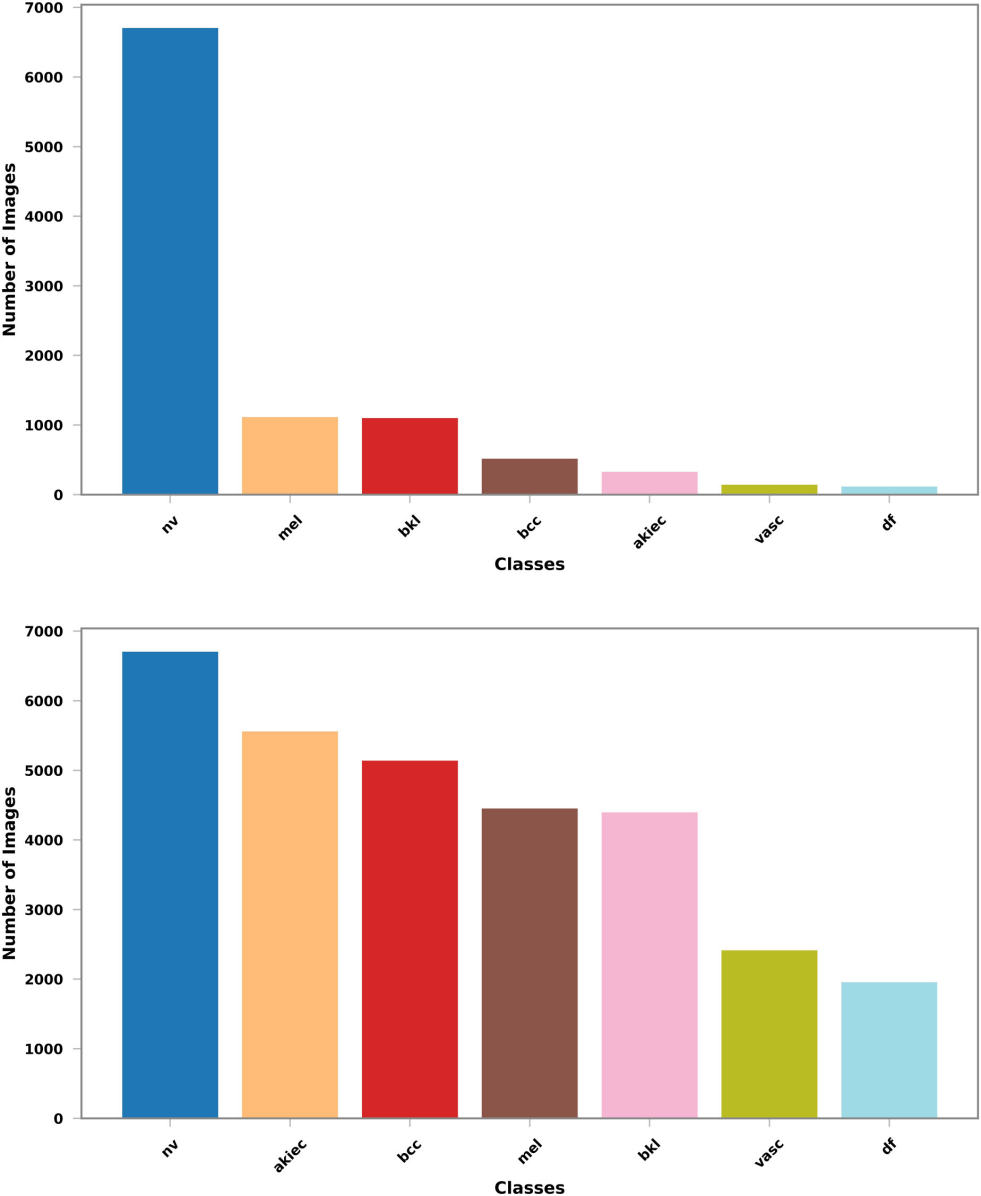
The class distribution of the human against machine with 10,000 training images (HAM10000) dataset: Left: Original dataset which is highly imbalanced, with a predominance of the ‘nv’ class; Right: Represents the dataset after applying augmentation techniques, which results in a more balanced distribution across different classes. This augmentation process helps to address the class imbalance and improve the performance of the model in skin cancer detection.

Class imbalances represent a challenge for classification and segmentation tasks alike. If left untreated, models may dedicate most of their capacities to the majority classes since the training loss is usually an unweighted sum over individual losses. Consequently, a class that has only half the samples of the other classes only contributes half as much to the loss and is often marginalized during the training process. Since different types of conditions have different prevalences, class imbalances are almost invariably present in medical data such as the images used in this work. To address the imbalance in the data set, we use techniques common in data augmentation: rotation by ±90°. and flips along the vertical and horizontal axes, as depicted in Figure 3. These operations are computationally cheap and preserve original input data samples (as opposed to transformations that rely on interpolation and resampling). Figure 6 summarizes the data set before and after augmentations. Note that, unlike some approaches, we do not use randomized oversampling. While it is easy to tweak randomized oversampling to yield a perfect 1 : 1 class balance, it hinders direct comparison between consecutive experiments as well as reproducibility, unless a fully augmented data set is shared for this purpose.

### Segmentation using UNet

After alleviating the class imbalance, Our segmentation framework is built on a UNet architecture that incorporates a VGG16 encoder, designed to process images of size 256 × 256 × 3 RGB from the ISIC 2018 dataset, which have been normalized to the [0–1] range. The encoder pathway features convolutional blocks from VGG16 with increasing filter sizes (64, 128, 256, and 512), along with max-pooling operations that systematically decrease spatial dimensions while enhancing the representation of features. The bottleneck layer utilizes 512 convolutional filters to effectively capture intricate lesion patterns.The decoder pathway employs up-convolutional (transpose convolution) layers alongside skip connections corresponding to the relevant encoder stages to enhance spatial resolution while preserving detailed information. The final layer of the network generates a 256 × 256 × 1 binary segmentation mask using sigmoid activation followed by thresholding.

**Figure 7.**
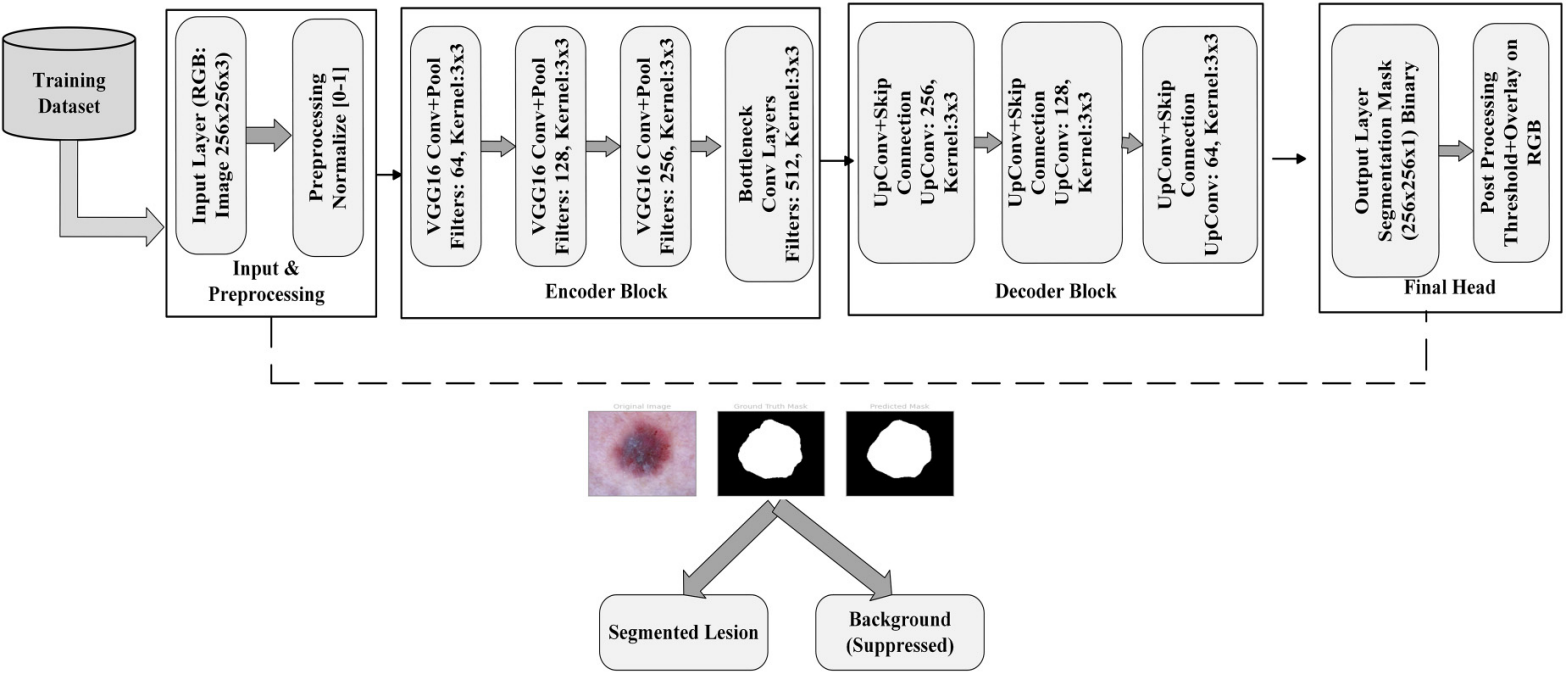
The U-Net architecture is utilized for the segmentation of skin lesions. It generates a binary mask that undergoes post-processing to apply thresholding and is projected onto the original RGB image.

During the post-processing phase, the binary mask is applied to the original RGB image to effectively isolate the lesion area while minimizing the visibility of the background tissue.This architecture achieves an optimal balance between feature extraction and spatial accuracy through the use of VGG16-based encoding blocks with 64, 128, and 256 filters, along with intelligent skip connections and accurate upsampling techniques. The concluding processing step, featuring RGB threshold-based visualization, guarantees precise identification of lesion boundaries while minimizing interference from adjacent tissue, which makes it well-suited for applications in dermatological image analysis. Figure 7 shows a architecture of UNet with VGG16 Encoder in detail form. The comprehensive design of U-Net combined with a VGG16 encoder is shown. Beginning with preprocessing and normalization, the diagram shows how the input image is processed through several steps. After that, it passes to the encoder block, which consists of convolutional layers with various kernel and filter sizes. The decoder block is then presented, which uses skip connections in conjunction with up-convolution layers to increase resolution. Finally, a binary mask generated by the output layer is subjected to additional processing for segmentation. By combining the segmentation skills of U-Net with the feature extraction power of the VGG16, this architecture allows for accurate skin lesion segmentation.

For this study, we considered UNet^58^ model for segmentation, and we present our comparison with state-of-art-models (SOTA) in Table 4.

**Table 4.** Comparison of the proposed framework to state-of-the-art models on the publicly available ISIC-2018 skin lesion dataset.

Method	Para (M)	FLOPs (G)	JI	DSC	ACC
FCN^61^	15.31	21.98	78.66	86.80	95.04
U-Net^29^	34.53	71.61	81.69	88.81	95.68
U-Net+^31^	36.63	151.59	81.87	88.93	95.68
AttU-Net^32^	34.88	72.81	81.99	89.03	95.77
DeepLabv3 + ^62^	39.76	47.34	82.32	89.26	95.87
DenseASPP^63^	35.37	42.63	82.53	89.35	95.89
BCDU-Net^64^	28.8	171.50	80.84	88.33	95.48
FocusNet-Alpha^65^	26.36	41.92	81.92	88.93	95.84
DO-Net^66^	24.75	122.45	82.61	89.48	95.78
CE-Net^24^	29.00	9.75	82.82	89.59	95.97
MultiResUNet^25^	7.25	20.52	82.25	89.15	95.90
CPFNet^26^	30.65	8.78	82.92	89.63	96.02
MFSNet^21^	30.59	8.02	82.95	89.82	95.99
MSCA-Net^23^	27.09	12.88	84.18	90.52	96.41
**Ours**	25.86	20.14	**89**.**12**	**94**.**24**	**97**.**59**

ISIC: International Skin Imaging Collaboration; JI: Jaccard index; DSC: Dice similarity coefficient; FLOPs: floating-point operations per second.

### Classification techniques

We employed two advanced transformer-based architectures, EfficientFormerV2 and SwiftFormer, to enhance skin lesion classification. EfficientFormerV2 leverages patch embedding, multi-head self-attention, and convolutional blocks to capture detailed information, while SwiftFormer utilizes MobileFormer blocks to strike a balance between computational efficiency and performance. Both models incorporate inverted residual blocks and efficient attention mechanisms to enhance feature extraction at various stages. Table 5 presents a comparison of these models’ performance on both unbalanced and balanced datasets, illustrating how balancing the data improves classification accuracy and F_1_ scores. The results underscore the effectiveness of transformer designs in tackling challenges related to class imbalance and dataset variability in lesion classification tasks.

**Table 5.** Classification accuracy and F1-score for different models on imbalanced and balanced datasets.

Model	Dataset	Accuracy (%)	*F*1-score (%)
EfficientFormerV2	Imbalanced	82.36	80.76
SwiftFormer	Imbalanced	78.63	77.39
EfficientFormerV2	Balanced	**97**.**11**	**97**.**14**
SwiftFormer	Balanced	**93**.**15**	**93**.**56**

### Performance measures

To assess how well models perform in skin lesion segmentation and classification, important performance metrics such as the Jaccard index (JI), Dice similarity coefficient (DSC), Accuracy (ACC), and F_1_-Score are commonly used. The JI quantifies the similarity between the predicted regions and the true ground truth areas, defined as follows: Given true positives (TPs) refer to the cases accurately recognized as positive, true negatives (TNs) refer to the cases accurately recognized as negative, false positives refer to the cases mistakenly classified as positive and false negatives refer to the cases mistakenly classified as negative. Additionally, recall is defined as the proportion of accurately predicted positive instances to all actual positive cases, whereas precision is defined as the proportion of accurately predicted positive instances to the overall number of predicted positive cases.

The **JI** evaluates the overlap between the anticipated and actual ground zones:

(1) $$\mathrm{JI}=\frac{\mathrm{TP}}{\mathrm{TP}+\mathrm{FP}+\mathrm{FN}}$$

The **DSC** examines how closely the anticipated segmentation matches the actual segmentation:

(2) $$\mathrm{DSC}=\frac{2\times \mathrm{TP}}{2\times \mathrm{TP}+\mathrm{FP}+\mathrm{FN}}$$

**ACC** indicates the portion of samples that were accurately classified:

(3) $$\mathrm{ACC}=\frac{\mathrm{TP}+\mathrm{TN}}{\mathrm{TP}+\mathrm{TN}+\mathrm{FP}+\mathrm{FN}}$$

The **F**_1_**-Score** provides a balanced evaluation of precision and recall through the use of their harmonic mean:

(4) $$F_{1}=\frac{2\times \mathrm{Precision}\times \mathrm{Recall}}{\mathrm{Precision}+\mathrm{Recall}}$$

## Experimental setup

The experimental setup was conducted on an Ubuntu 22.04 system, equipped with a robust configuration including 512GB of RAM, an Intel Xeon(R) Gold 6226R processor, and an NVIDIA RTX 3090 graphics processing unit (GPU) featuring 24GB of Video Random Access Memory. The framework's implementation was executed through Python in Jupyter Notebook, leveraging the Tensorflow library to facilitate comprehensive training and testing capabilities on the GPU.

For the image segmentation task, we used a VGG16 encoder integrated with a U-Net framework, trained on the ISIC-2018 dataset.^14^ The model underwent training for 100 epochs at a learning rate of 10*^−^*^4^ and a batch size of 32. We applied the binary cross-entropy loss function and kept track of the validation loss during the training procedure. The ISIC-2018 dataset consists of 2,594 RGB images and is a well-known standard for assessing medical imaging algorithms. For our experiments, every image was resized to a dimension of 224 × 224 pixels, and the dataset was divided into training, validation, and test sets following a 70%–10%–20% ratio based on MSCA-Net.^23^

## Results

The main goal of this research is to create a solid framework that combines deep CNN-based segmentation with ViT models for accurate classification of skin lesions. We utilized the ISIC 2018 dataset and HAM10000, which featured various types of skin lesions. For accurate segmentation of lesions, we employed U-Net with a VGG16 encoder, while the EfficientFormerV2 and SwiftFormer models were applied for classification tasks. The datasets underwent preprocessing and were resized to 256 × 256 × 3 to ensure uniform input dimensions across all models. This two-step approach enhances both segmentation and classification performance, effectively addressing the challenges posed by imbalanced data.

### Segmentation

**Figure 8.**
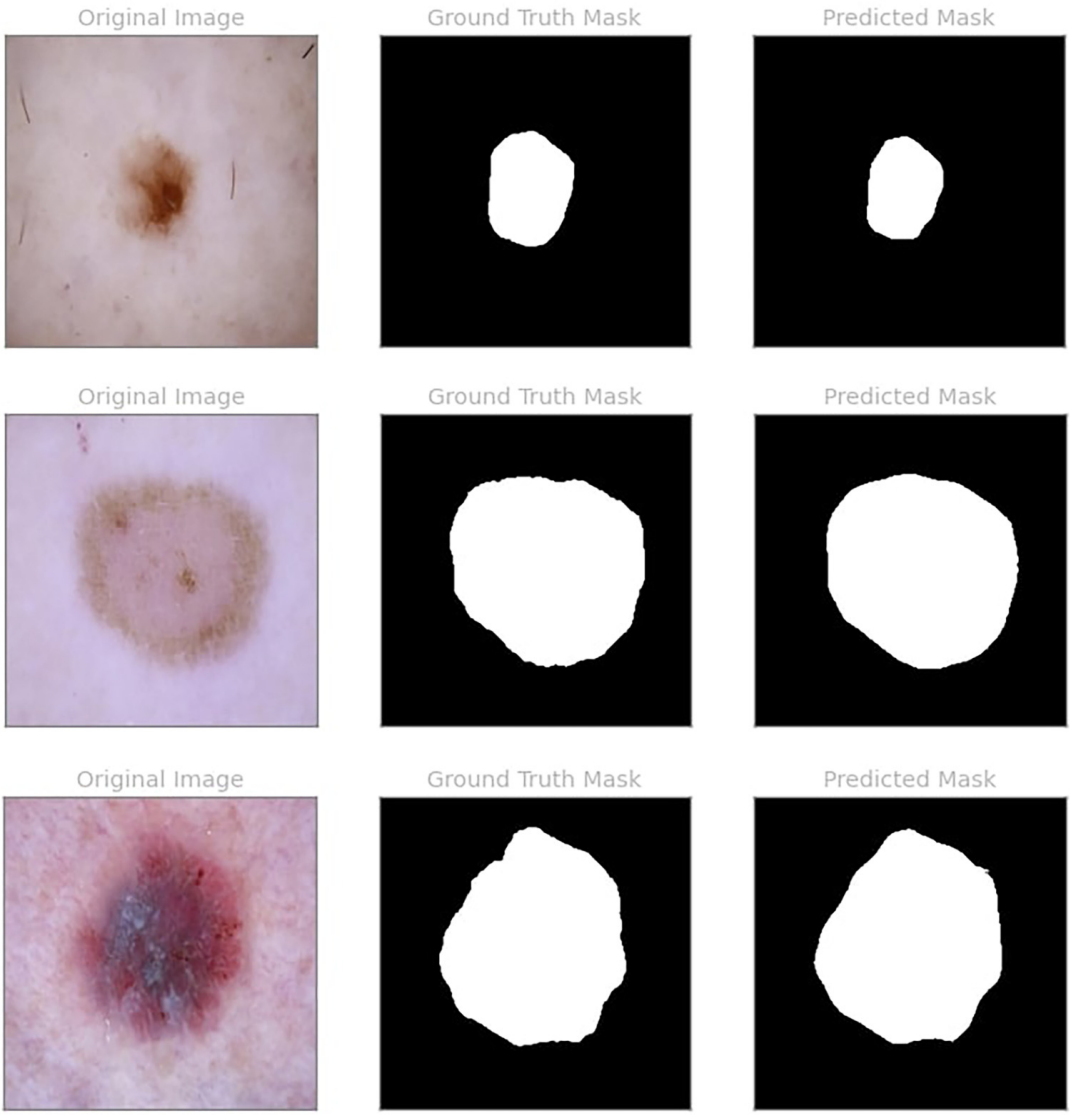
Segmentation visual results: Predictions of U-Net segmentations on International Skin Imaging Collaboration (ISIC)-2018 Dataset.

We conducted a comprehensive segmentation task using the ISIC 2018 skin lesion dataset, employed a U-Net architecture with a VGG16 encoder. Our primary objective was to assess the impact of balancing the dataset on segmentation accuracy. The results from our experiments are summarized in Table 4, and Figure 8 illustrates the segmentation visual results, where the first image represents the original skin lesion, the second demonstrates the ground truth, and the third represents the predicted mask. Table 4 presents a comparison of our suggested framework with multiple leading models using the ISIC-2018 skin lesion dataset. The criteria utilized for comparison consist of the total number of parameters (Para), floating-point operations per second (FLOPs), JI, DSC, and accuracy.

Our proposed model demonstrates superior performance compared to current leading models across multiple evaluation metrics (all in %). Specifically, our approach achieves a JI of 89.12, a DSC of 94.24, and an accuracy of 97.59. These results represent significant improvements over previous top-performing models, such as MSCA-Net, which previously recorded a JI of 84.18, a DSC of 90.52, and an accuracy of 96.41, highlighting the substantial advancements of our proposed methodology.

### Classification on the HAM10000 dataset

We performed the classification task on the HAM10000 dataset utilizing a ViT. The dataset exhibits a significant imbalance, particularly with the ‘nv’ class being much more prevalent than the others. To tackle this discrepancy, we implemented data augmentation strategies to create more images for the underrepresented classes.

We assessed our proposed framework using both imbalanced and balanced versions of the dataset across various models, including Efficientformer and Swiftformer. The Table 5 displays the classification accuracy and F_1_-score for the different models.

Also, we assess the performance on HAM10000 dataset, Table 6 present SOTA comparison of our findings with various leading models on the HAM10000 dataset. The outcomes from our balanced dataset show notable advancements in both accuracy and F_1_-score, highlighting the success of our augmentation strategy.

**Table 6.** Comparison of previous studies on the HAM10000 dataset.

Models	Accuracy (%)	*F*1-score (%)
DenseNet201^67^	82.9	74.4
Wide-ShuffleNet^68^	86.3	—
Collective Intelligence-based System^69^	86.7	—
MobileNet V2-LSTM^70^	90.7	—
Mask-RCNN^71^	86.5	86.2
EfficientNet-B4^72^	85.8	—
KELM classifier^73^	90.6	—
DenseNet201^74^	87.7	85.5
CRCKD algorithm^75^	85.6	76.4
MobileNet^76^	83.1	83.0
Deep CNN^77^	84.0	—
L2 regularisation^78^	72.1	—
Alam et al.^20^	91.0	88.1
**Our study (EfficientFormerV2)**	**97.11**	**97.14**

HAM10000: Human against machine with 10,000 training images; CNN: convolutional neural network.

## Results on SLICE-3D dataset

The SLICE-3D^57,79^ dataset presents a real-world binary classification task: predicting the probability of malignancy from cropped lesion images. This data set is very challenging since it was imaged using 3D-TBP, which, unlike dermoscopy, is a relatively new modality. While 3D-TBP offers potential advantages such as image ortho-rectification, that is, removing image distortions due to body curvature, it also comes with a distinct disadvantage: methods that work exceptionally well for dermoscopic images do not necessarily work for 3D-TBP. In addition, combining dermoscopic and 3D-TBP data to augment the amount of data does not necessarily result in models with higher performance. We explored two modeling strategies:

### Strategy I—tabular-only classifier

In the first approach, we trained a classifier using only the structured metadata provided with each lesion. This includes demographic, anatomical, and geometric lesion descriptors. The goal was to establish a fast and interpretable baseline that could work without visual data.

**Model:** XGBoost classifier

**Features:** Age, sex, anatomical site, lesion size, LAB* color features (e.g., tbp lv deltaLBnorm), symmetry, nevi/melanoma confidence scores, and 3D lesion coordinates (tbp lv x/y/z).

**Preprocessing:** Missing value imputation (mean/mode), one-hot encoding for categorical variables, feature scaling (standard scaling)

**Training:** Binary classification using log-loss and early stopping, with stratified train-validation splits. Final predictions were submitted as a CSV file with isic id and predicted probability of malignancy.

This approach achieved a **public leaderboard score of 0.16752** based on the partial area under the receiver operating characteristic curve (pAUC) above 80% true positive rate (TPR).

### Strategy II—image + tabular fusion classifier

In the second approach, we enhanced predictive performance by combining deep image features with tabular metadata, better simulating real-world clinical decision-making.

**Image encoder:** Pre-trained ResNet18 (ImageNet backbone), final fully connected layer removed for 512-dimensional feature extraction, images resized to 224 *×* 224 and normalized using ImageNet statistics

**Tabular branch:** Same feature selection and preprocessing as Strategy I.

**Fusion strategy:** Concatenation of image and metadata feature vectors, passed through fully connected layers with ReLU activation and Dropout. A final sigmoid layer outputs the malignancy probability.

**Training:** Weighted sampling to address class imbalance, optimized using Adam with learning rate scheduling and binary cross-entropy loss. Models were validated using the competition-specific pAUC metric.

This multi-modal approach yielded a **public leaderboard score of 0.15792** and demonstrated improved generalization on rare malignant cases.

### Evaluation metric

The competition uses a custom metric: pAUC above 80% TPR, emphasizing clinical utility by focusing on high-sensitivity regions of the receiver operating characteristic (ROC) curve. The score is computed as:

$${\mathrm{pAUC}}_{>0.8}=\int_{0.8}^{1.0}\mathrm{TPR}(\mathrm{FPR})\mathrm{dFPR}$$

The maximum achievable score is 0.2. We show results of SLICE-3D^57,79^ in Table 7 for both strategies (i.e., Tabular-only (metadata) and Images + Tabular) data.

**Table 7.** Kaggle submission scores on SLICE-3D dataset.

Method	Public Score (pAUC)	Description
Tabular-only	**0**.**16752**	XGBoost on structured metadata
Image + Tabular	**0**.**15792**	ResNet18 + tabular feature fusion

pAUC: partial area under the receiver operating characteristic curve.

**Figure 9.**
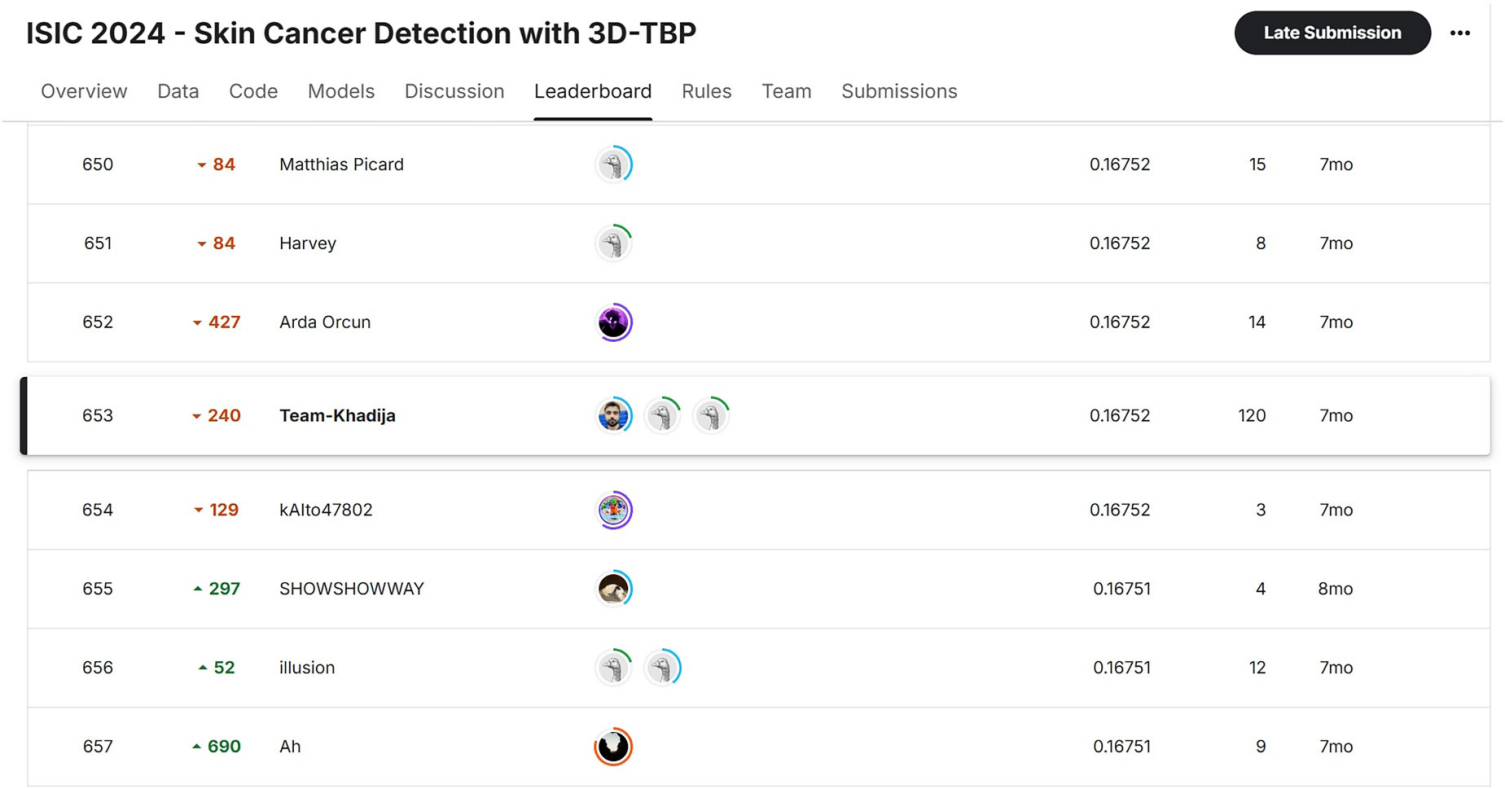
Leaderboard excerpt showing Team-Khadija's final placement (Rank: 653).

***Final leaderboard ranking*** Out of over 1300 teams, we ranked **653** on the final private leaderboard with a private score of **0.16752**, placing us in the upper mid-tier of submissions. Our consistent improvements across versions are visualized in Figure 9.

## Discussion

The results of this study highlight the potential of our two-phase approach for melanoma detection in skin lesion segmentation and classification. Our framework effectively addresses key challenges in automated diagnosis, particularly class imbalance, lesion heterogeneity, and real-world deployment constraints. Using a U-Net architecture with a VGG16 encoder, our segmentation model achieved a DSC of 94.24%, a JI of 89.12%, and segmentation accuracy of 97.59% on the ISIC 2018 dataset.

To address classification, we employed EfficientFormerV2 and SwiftFormer on the HAM10000 dataset. After class balancing via data augmentation, EfficientFormerV2 achieved 97.11% accuracy and a 97.14% F_1_-score. These improvements confirm the importance of handling class imbalance in clinical datasets where rarer conditions such as melanoma are underrepresented.

To validate the generalizability of our approach on a large-scale and clinically realistic dataset, we also evaluated on the recently introduced ISIC 2024 SLICE-3D challenge dataset. This dataset contains over 400,000 lesion crops from 3D-TBP and represents both strongly-labeled (biopsy-confirmed) and weakly labeled (clinically benign) examples. On this dataset, our tabular-only XGBoost model achieved a pAUC score of 0.16752, while the image + tabular fusion model scored 0.15792. These results demonstrate competitive performance in highly imbalanced, non-dermoscopic settings and affirm the adaptability of our framework beyond traditional dermoscopic environments.

The proposed approach shows considerable benefits due to its high precision and effective management of class imbalance. By utilizing strong data balancing strategies and sophisticated model architectures, the framework achieves remarkable enhancements in both segmentation and classification performance. This integrated segmentation and classification capability highlights its significant potential for application in clinical settings, aiding dermatologists in accurately diagnosing skin cancer. In addition, the model's versatility suggests its usefulness in various medical image analysis tasks, opening avenues for future exploration in different healthcare fields. These findings emphasize the necessity of incorporating data augmentation, balanced datasets, and powerful deep learning architectures to improve the precision, dependability, and real-world implementation of automated skin cancer detection systems, ultimately enhancing diagnostic tools in medical environments.

Despite these promising outcomes, several challenges persist. First, segmentation performance may degrade on atypical or small lesions, and on poor-quality input images. Second, real-time deployment in teledermatology settings is hindered by the computational complexity of transformer-based models such as EfficientFormer and SwiftFormer. Although models like LightCF-Net^34^ prioritize real-time inference through lightweight architectures. Future research could explore hybrid architectures that blend our method's robustness with the real-time capabilities demonstrated by LightCF-Net. Third, performance may vary on unseen domains due to distributional shifts in patient populations, lighting conditions, and acquisition modalities.^34^

To overcome these limitations, future work should prioritize lightweight architectures for edge deployment, model optimization for speed without loss of accuracy, and domain generalization techniques to ensure cross-population robustness. Integrating patient context and temporal lesion monitoring into model inputs could further enhance diagnostic reliability. Finally, additional validation on diverse skin tones and populations especially underrepresented demographics will be essential to ensure equity in clinical deployment.

## Conclusion

This research assessed skin lesion segmentation and classification using the ISIC 2018 and HAM10000 datasets. For segmentation, a U-Net model with a VGG16 encoder achieved strong results, with a JI of 89.12%, a DSC of 94.24%, and an Accuracy of 97.59%. In classification, addressing class imbalance through data augmentation significantly improved performance, with the EfficientFormerV2 model reaching 97.11% accuracy and a 97.14% F_1_-score on the balanced data, outperforming results on the original imbalanced data. These findings highlight the effectiveness of combining data balancing with advanced deep learning architectures for skin lesion analysis and suggest strong potential for clinical translation, especially in resource-constrained environments and for improving diagnostic accuracy in telemedicine and mobile skin cancer detection. This work contributes to the development of more reliable and scalable automated skin cancer identification systems.

## A word of caution

While this study demonstrates that our framework has the potential to be used for self-assessment and diagnosis, it is a technical study. Further clinical studies and a careful risk assessment of its impact on patients’ wellbeing as well as the healthcare system is needed before deploying it at scale.
